# Accounting for variation in designing greenhouse experiments with special reference to greenhouses containing plants on conveyor systems

**DOI:** 10.1186/1746-4811-9-5

**Published:** 2013-02-08

**Authors:** Chris J Brien, Bettina Berger, Huwaida Rabie, Mark Tester

**Affiliations:** 1 University of South Australia, GPO Box 2471, Adelaide, SA 5001, Australia; 2 University of Adelaide, PMB 1, Glen Osmond, SA, 5064, Australia

**Keywords:** Automated phenotyping, Conveyor system, Greenhouse experiments, Greenhouse experimental design, Microclimate variation, Plant relocation, Statistical analysis, Thigmomorphogensis

## Abstract

**Background:**

There are a number of unresolved issues in the design of experiments in greenhouses. They include whether statistical designs should be used and, if so, which designs should be used. Also, are there thigmomorphogenic or other effects arising from the movement of plants on conveyor belts within a greenhouse? A two-phase, single-line wheat experiment involving four tactics was conducted in a conventional greenhouse and a fully-automated phenotyping greenhouse (Smarthouse) to investigate these issues.

**Results and discussion:**

Analyses of our experiment show that there was a small east–west trend in total area of the plants in the Smarthouse. Analyses of the data from three multiline experiments reveal a large north–south trend. In the single-line experiment, there was no evidence of differences between trios of lanes, nor of movement effects. Swapping plant positions during the trial was found to decrease the east–west trend, but at the cost of increased error variance. The movement of plants in a north–south direction, through a shaded area for an equal amount of time, nullified the north–south trend. An investigation of alternative experimental designs for equally-replicated experiments revealed that generally designs with smaller blocks performed best, but that (nearly) trend-free designs can be effective when blocks are larger.

**Conclusions:**

To account for variation in microclimate in a greenhouse, using statistical design and analysis is better than rearranging the position of plants during the experiment. For the relocation of plants to be successful requires that plants spend an equal amount of time in each microclimate, preferably during comparable growth stages. Even then, there is no evidence that this will be any more precise than statistical design and analysis of the experiment, and the risk is that it will not be successful at all. As for statistical design and analysis, it is best to use either (i) smaller blocks, (ii) (nearly) trend-free arrangement of treatments with a linear trend term included in the analysis, or, as a last resort, (iii) blocks of several complete rows with trend terms in the analysis. Also, we recommend that the greenhouse arrangement parallel that in the Smarthouse, but with randomization where appropriate.

## Background

Two competing approaches for dealing with microclimate variation in the design of greenhouse experiments are:

1. Place the experimental material in a convenient location in the greenhouse and then re-arrange the relative locations of plants in a haphazard manner throughout the experiment.

2. Employ an experimental design that keeps the plants in the same relative positions throughout the experiment and then use a statistical analysis to adjust for microclimate, and other, differences.

The justification for the first approach is that the rearrangement will even out the plants by exposing all plants to a range of the microclimates occurring in the greenhouse in which the experiment is conducted (see for example [1]). The disadvantages are listed in [2] as being the labour involved, the possibility of injury to plants, and the opportunity for unobserved biases. The latter relates to the possibility that not all plants will be equally exposed to the different microclimates that occur because, generally, there is no defined process to ensure that this is the case. A mechanical rotation system for reducing the labour required is described in [1]. It is speculated in [3] that, provided the possibility of plant injury could be avoided, then there could be substantial improvements in precision, provided that an assumed decrease in variability due to location eventuates. Reduced variability in rice grown in pots on a continuously rotating platform was reported in [4], and so that experiments run using this system would have better precision than pots in fixed positions on benches. Another advantage of moving the plants is the potential for a thigmomorphogenic effect [5] that would result in shorter, thicker plants. Given that plants normally grow in fields, with wind moving them, possible thigmomorphogenic effects from movement in the greenhouse could lead to plants having growth more like that found in field-grown plants. On the other hand, there is also the possibility of soil compaction due to the movement of pots on the belt which could potentially have adverse effects on plant growth [6]. It is our experience that excessive soil compaction can occur when the soil in the pots on the belt have a very high clay content. Similarly, we have found that substrates with a very high sand content are not suitable for conveyor experiments due to soil shifting in the pots on the belt and roots being damaged as a result.

The justification for the second approach is that major differences in microclimate experienced by the plants, resulting in what can be termed global variation, can be accounted for in the experimental design and adjusted for in the statistical analysis. Some references in which the use of designs with rows and columns for experiments in greenhouses is recommended to achieve this are [7,8]p. 117] and [9-11]. Often the plants in greenhouse experiments are arranged in square or rectangular grids and such designs will deal with trends in the north/south direction that might be caused by the changing angle of the sun during the growing season and also trends in the east/west direction caused by difference in microclimate experienced by the plants during a day.

Another possibility is that spatial designs might be employed to take account of the tendency for neighbouring plants to be similar that results in small-scale trends in variation, referred to as local spatial variation. Some evidence for the need to account for local spatial variation comes from [12], in which small-scale spatial variability in photosynthetically active radiation in a gable-roof greenhouse is demonstrated. Spatial designs have been recommended for field trials to deal with such variation [13] and so one might do the same for greenhouse experiments.

It was decided to investigate these issues in designing greenhouse experiments in the context of The Plant Accelerator^®^ (PA) at the Australian Plant Phenomics Facility in South Australia [14,15]. This facility consists of four Smarthouses and 34 conventional greenhouses. The technologically advanced Smarthouses utilize the LemnaTec-Scanalyzer 3D platform [16] and are fully climate-controlled greenhouses equipped with computer-controlled conveyor belts carrying up to 600 plants per room. Plants are carried on this conveyor system in individual carts for regular imaging, weighing and watering. There is the possibility that this movement may have a thigmomorphogenic or other effect. As well as managing plant movement and tracking, the conveyor system allows for plant locations to be rotated during an experiment. The facility is aligned on a north/south axis and so a trend from south to north can be expected due to changes in the angle of the sun. Also, both the Smarthouses and the greenhouses have air conditioners, usually along one side and so a trend away from the air conditioners can be expected. The primary response measured on plants is the total area exhibited in three images, this being related to plant biomass [17].

**Figure 1:**
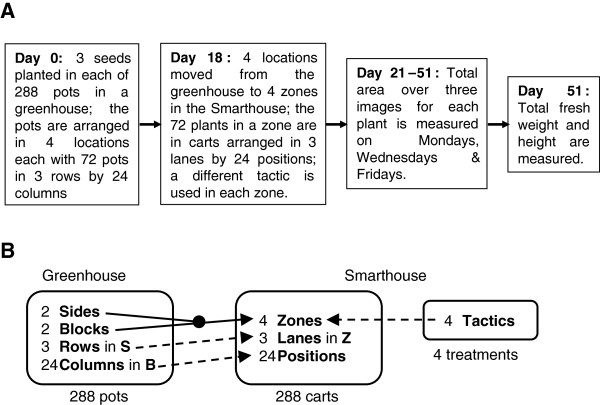
**Overview (A) of and factor allocation diagram (B) for The Plant Accelerator^®^ (PA) experiment. **The factor allocation diagram shows how pots and treatments were allocated to carts. The solid arrow indicates that the allocation was done by randomization and the two lines leading to the sold black circle that it was the combinations of the factors to the left that was randomized; a dashed arrow indicates that the allocation of one factor to another was systematic. (S = Sides, B = Blocks and Z = Zones.)

We conducted an experiment, the PA experiment, an overview of which is given in Figure 1, along with a factor allocation diagram [18] showing the assignments of the factors to each other; full details are in the Methods section. In this experiment, plants of a single line of wheat were housed in a (conventional) greenhouse within the facility for germination and initial growth. Here, they were in pots arranged in 6 rows by 48 columns. After 18 days they were transferred to the Smarthouse for the remainder of the experiment, where the pots were placed in carts arranged in 4 zones each of which initially consisted of 3 lanes by 24 positions. Thus the experiment is two phase [19]: a first phase in a greenhouse and a second phase in a Smarthouse. In the Smarthouse phase, the plants in a zone were subjected to one of the following four tactics:

1. **Bench**: the plants were placed on fixed benches located alongside the conveyor system at its southern edge. These plants were weighed and watered manually and were always replaced in the same positions on the benches.

2. **Same lane**: the plants were placed in lanes 1–3 in the Smarthouse. These plants were always returned to the same positions after imaging/watering.

3. **Half lane**: the plants were placed initially in lanes 4–6 in the Smarthouse. After imaging/watering, these plants were moved forward a half lane so that the 12 carts in the western half of a lane were moved to the eastern half of the same lane and the 12 carts in the eastern half of a lane were moved to the western half of the next lane. Once carts had occupied the eastern half of lane 11, they were next moved back to the western half of lane 4.

4. **Next lane**: the plants were initially placed in lanes 12–14 in the Smarthouse. After imaging/watering, these plants were moved forward to the lane next to the one from which they had come. Once a lane of plants had been in lane 24, they were next moved back to lane 12.

That is, each tactic was applied to 72 carts initially arranged in 3 Lanes by 24 Positions in the Smarthouse.

Same lane is the standard tactic for glasshouses with this system. The bench tactic corresponds to traditional greenhouse practice, when pots are not relocated. It was included in order to compare it with the same-lane tactic so that the effect of the movement of carts in the Smarthouse could be assessed. The half-lane and next-lane tactics represent relocation of the carts across the directions in which the major trends are expected during the Smarthouse phase. Differences in the analysis results between these two tactics and the same-lane tactic will provide an evaluation of the strategy of relocating plants during an experiment, as opposed to statistically adjusting for trends.

The results of this experiment are analysed to establish the important sources of variation in such experiments, although it is not possible to use them to study variation across the full set of 24 lanes in the Smarthouse. For this latter aspect, the results of three multiline experiments, described in the Methods section, are used. We will also use the results of the PA experiment to examine the effect of movement in the Smarthouse and the effectiveness of the relocation strategies. Finally, an investigation of alternative statistical design and analysis strategies for greenhouse experiments will be examined using the data from the PA experiment. Because the plants in a tactic are from a single line, this data is well suited to such a study.

## Results

The main response is total area of the plants measured between 21 and 51 days after planting, although only that for 51 days after planting is available for the bench tactic. In addition, shoot fresh weight, height and a density index at 51 days after planting are reported. Hereafter, days after planting will simply be referred to as ‘day’. Plots of the raw data are available (see Additional file 1). They show the variability in the responses and the evidence for trends in the data.

### Sources of variation and tactic differences in the PA experiment

Mixed models were fitted, as described in the Methods section, to all the response variables from the PA experiment. The results of these analyses are given in Table 1. It is noted that the autocorrelation terms are fitted to assess the presence of local spatial variation. All the other terms tested represent various forms of global variation, except for heterogeneous variances and for the terms with just Locations or Tactics.

**Table 1:** Results of hypothesis tests from the mixed model analyses for all response variables

	**Day 21**		**Day 51**							
	**Total area**		**Total area**		**Fresh weight**		**Height**		**Density index**	
	***P***^**b**^	**Action**^**c**^	***P***^**b**^	**Action**^**c**^	***P***^**b**^	**Action**^**c**^	***P***^**b**^	**Action**^**c**^	***P***^**b**^	**Action**^**c**^
**Random model change**^**a**^										
Add heterogeneous *Locations */ *Tactics* variances^d^	0.023	Retain	0.021	Retain	0.061	Retain	0.017	Retain	0.161	Retain
Add *columns* / *Positions *ar1, differing for		Omit		Omit		Omit		Retain		Omit
*Locations* / *Tactics*										
Drop *Columns∧Locations* /		Omit		Omit		Omit		Omit		Omit
*Positions∧Tactics *deviations										
Drop *spl(Columns)∧Locations/*		Retain		Omit		Retain		Retain		Omit
*spl(Positions)∧Tactics*										
Drop *position *deviations			DNF	Omit	DNF	Omit	0.120	Omit	DNF	Omit
Drop *spl(Position)*			DNF	Omit	DNF	Omit	DNF	Omit	DNF	Omit
Check heterogeneous *Locations */ *Tactics *variances^d^	0.038	Retain	0.015	Retain	0.025	Retain	0.007	Retain	0.392	Omit
Check ar1 on *Columns */ *Positions*, differing for		Omit		Omit		Omit		Omit		Omit
*Locations */ *Tactics*										
**Fixed model testing**^**a**^										
*lin(Columns)∧Locations */ *lin(Positions)∧Tactics*	<0.001	NA	0.026	Retain	0.036	NA	0.005	NA	0.012	Retain
lin(Positions)			<0.001	NA	<0.001	NA	0.710	NA	<0.001	NA
*Locations∧Rows */ *Tactics∧Lanes*	0.210	ns	0.405	ns	0.311	ns	0.311	ns	0.772	ns
*Locations */ *Tactics*	0.099	NA	<0.001	NA	<0.001	NA	0.007	NA	<0.001	NA

^a^The italicized term before the slash is for Day 21 and the one after the slash is for Day 51.

^b^DNF indicates that the parameters in the changed model “Did Not Fit”, either because of a lack of convergence or because the estimates for one or more parameters was zero; on occasion terms with a *P*-value between 0.05 and 0.10 are retained on the grounds that there is some indication that the terms are required.

^c^NA indicates that testing was not applicable because, for this term, (i) its factors are a subset of those for a significant fixed term or (ii) it has the same factors as a higher-order term that is significant; ns indicates not significant.

^d^A significance level of 0.25 is used for this hypothesis test in order to decrease the chance of a Type I error and to err on the side of allowing for heterogeneous variances if there is some evidence for them.

**Figure 2:**
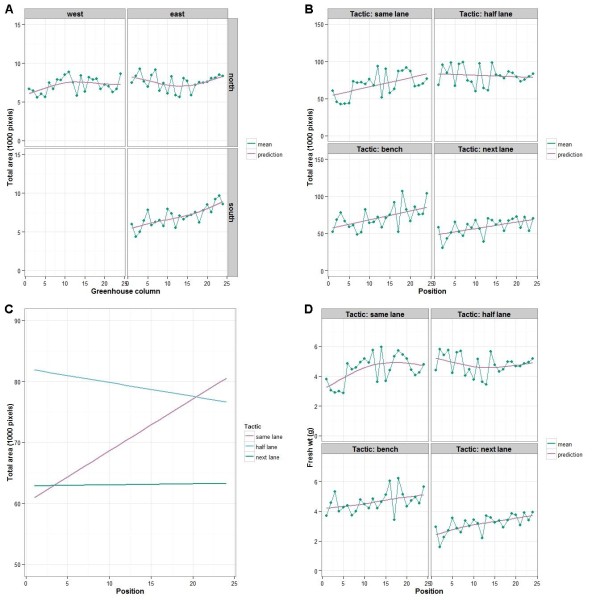
**Fitted trends over columns and positions.** (**A**) with column means, for total areas on day 21; (**B**) with position means, for total areas on day 51; (**C**) for total areas on day 51, adjusted for total area on day 21; (**D**) with position means, for fresh weight on day 51. Each plot in (**A**) is for a location in the greenhouse and in (**B**) and (**D**) for a zone in the Smarthouse, with the arrangement of the plots mirroring their geographical location in the greenhouse.

The results for the total areas on day 21, which reflect the variation that had arisen in the greenhouse phase, show that Locations differ in their variances and that there is a curvilinear trend over Columns that differs between Locations. To examine in more detail the sources of variation that the mixed model fitting indicates are present in the total areas on day 21, the predicted averages at the centre of a Row, along with their standard errors, and the coefficients of variation (CVs) are given for each location in Table 2. The predicted average for the south-east location is close to being significantly less than that for the north-west location, but appears to have higher variance than the other locations. Plots displaying the Column trend are in Figure 2A. They show a flat undulating trend on the northern side and a more pronounced increasing trend in the south-east.

**Table 2:** Summary of differences between locations or tactics for all response variables

**Day 21**			**Location**		
			**North-west**	**North-east**	**South-east**
Total area	Predicted average^ab^		7.562	7.031	6.828
(1000 pixels)	Standard error^c^		0.2558	0.2786	0.3153
	CV (%)^d^		19.3	20.3	25.8
**Day 51**		**Tactic**			
		**Bench**	**Same lane**	**Half lane**	**Next lane**
Total area	Predicted average^ab^	71.62	68.90	80.78	59.06
(1000 pixels)	Standard errors ^c^	2.383	2.421	2.711	1.827
	CV (%)^d^	28.2	29.8	28.5	26.3
Fresh weight	Predicted average^ab^	4.626	4.784	4.591	3.207
(g)	Standard errors ^c^	0.1803	0.1946	0.2040	0.1523
	CV (%)^d^	22.7	24.3	26.9	26.4
Height	Predicted average^ab^	37.79	39.01	38.30	37.01
(cm)	Standard errors ^c^	0.567	0.493	0.649	0.663
	CV (%)^d^	8.9	7.2	10.5	11.2
Density index	Predicted average^ab^	1.931	1.812	2.109	1.646
(1000 pixels / cm)	Standard errors ^c^	0.068			
	CV (%)^d^	30.9			

^a^An approximate LSD (5%) for total area on day 21 is 0.804; an approximate LSD (5%) for total area on day 51 is 6.67, for fresh weight is 0.520 and for height is 1.69; an exact LSD for the density index is 0.193.

^b^The predictions are for the average value at the centre of a row or lane.

^c^The standard errors give an indication of the variability of the predicted averages, but LSDs are required to judge whether pairs of predicted averages are different. The standard errors are proportional to the fitted standard deviations for the different locations/tactics.

^d^The coefficients of variation (CV) measure the variability of individual plants.

For all the response variables on Day 51, except Density, the variances differ between Tactics. All variables show a smooth trend over Position with the trend being linear for total area and density index and curvilinear for shoot fresh weight and height.

To examine in more detail the sources of variation that the mixed model fitting indicates are present in the day 51 response variables, the predicted averages at the centre of a Lane, along with their standard errors, and the CVs are given for each tactic for all response variables in Table 2. Plots displaying the Position trend for total areas and for fresh weights are in Figures 2B and 2D, respectively.

For the total areas on day 51, the predicted average for the next-lane tactic is significantly less than that for the bench and same-lane tactics, and all of these are significantly less than the half-lane tactic. It would appear that the next-lane tactic is less variable than the other tactics. The Position trend, in Figure 2B, is an increasing trend from west to east for all tactics, except for the half-lane tactic, which is flat. A supplementary hypothesis test shows that the linear trend does not differ significantly (*P* = 0.7260) between the bench, same-lane and next-lane tactics.

For fresh weights on day 51, the predicted average for the next-lane tactic is significantly less than that for the other tactics, none of the other tactics being significantly different. It would appear that the next-lane tactic is less variable than the other tactics. The Position trend, in Figure 2D, is an increasing trend from west to east for all tactics, except for the half-lane tactic, which is flat. The trends in the total area are consistent with those in fresh weight.

For height on day 51, the predicted average for the next-lane tactic is significantly less than that for the same-lane tactic, but none of the other tactics are significantly different. While the addition of autocorrelation to the model was initially significant for this response variable, the estimated values of the correlation coefficients for the tactics are –0.361, 0.331, –0.215 and 0.004, respectively. These indicate that the autocorrelation is, at best, weak and in the end is not significant (see Table 1).

For the density index on day 51, plants from the next-lane tactic are on average less dense than those for the bench and half-lane tactics; the plants for the same-lane tactic are intermediate between them, not being significantly different from any of the other tactics (*P* > 0.05).

### Adjusting total area on day 51 for total area on day 21 in the PA experiment

An analysis of covariance examined how total areas on day 51 are related to total areas on day 21 by using linear and spline terms for day 21 as covariate terms. It showed that, while the curvature in the relationship, as measured by a spline term, does not differ significantly between tactics (the estimate for this term was zero), there is significant curvature common to all tactics (*P* = 0.002). On the other hand, differences between tactics in the linear relationship are effectively significant (*P* = 0.051). Having adjusted for the relationship with total areas on day 21, it was found that (i) the linear trend over Positions differs significantly between Tactics (*P* = 0.015), (ii) there are no differences between Lanes within Tactics (*P* = 0.197), and (iii) Tactics differ significantly (*P* < 0.001). The predicted averages at the mean for total area on Day 21, along with their standard errors, and the CVs are given for each tactic in Table 3. The CV is between 7% and 10% less as a result of the inclusion of the total area on day 21 as a covariate. There is very little difference between the variance for same and half- lane tactics. The fitted linear trend over Positions for the total area on day 51 adjusted for the total area on day 21 is given in Figure 2C. It shows that only for the same-lane tactic is the trend in adjusted total area across the positions not flat.

**Table 3:** Summary of differences between tactics for the total area on day 51, after adjustment for the total area on day 21

		**Tactic**	
	**Same lane**	**Half lane**	**Next lane**
Predicted average^ab^	70.76	79.29	63.08
Standard errors^c^	2.037	2.129	1.570
CV (%)^d^	22.5	20.5	16.6

^a^An approximate LSD (5%) is 2.46.

^b^Each prediction is for the average value at both (i) the centre of a lane and (ii) the mean value for total area on day 21.

^c^The standard errors give an indication of the variability of the predicted averages, but LSDs are required to judge whether pairs of predicted averages are different. The standard errors are proportional to the fitted standard deviations for the different tactics.

^d^The coefficients of variation (CV) measure the variability of individual plants.

### The effect of separating the Position trend on the precision of total area on day 51 in the PA experiment

To examine the effect on the precision of an experiment of not isolating Position trend, we have fitted two sets of separate mixed models to each tactic. In the first set, the model for a tactic has Lane and Position terms and in the second set it has just a Lane term. The precision is measured using the standard deviations $\left(=\sqrt{\text{error}\;\text{variance}}\right)$ and their values are in Table 4; also shown are the relative precisions that are calculated as the ratios of the variance when Positions are pooled with the error variance to that for when Positions are separated.

**Table 4:** Standard deviations for total area on day 51 for each tactic with and without Positions pooled

	**Standard deviation (1000 pixels)**		**Relative precision (%)**
**Tactic**	**Positions separated**	**Positions pooled**	
Bench	18.9	21.8	133.0
Same lane	19.0	22.2	137.0
Half lane	23.7	22.9	92.8
Next lane	14.9	16.6	122.9

For all, except the half-lane tactic, separating Positions from the error variance increases the precision by as much as 37%. In the case of the half-lane tactic, there is a small decrease. It is of note that the bench and same-lane standard deviations are increased to a value nearer that for half-lane. That is, the magnitude of the half-lane error variance is consistent with being inflated by an amount equivalent to that resulting from position differences of the magnitude observed in this experiment.

### Growth trend for different tactics in the PA experiment

**Figure 3:**
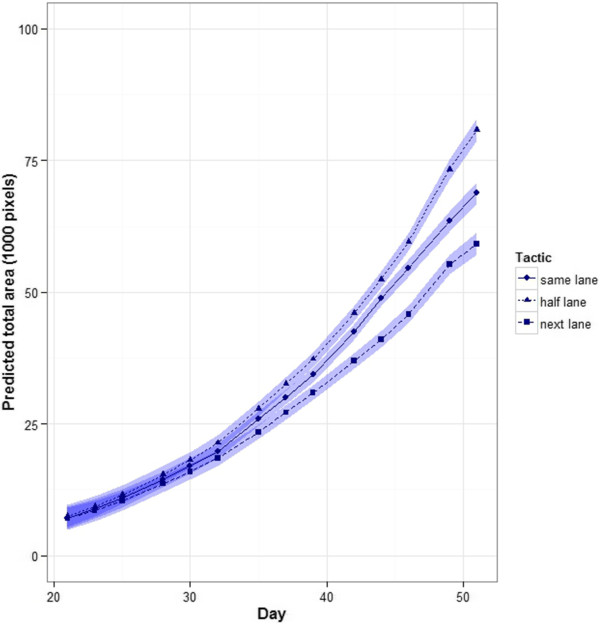
**Trend in total area over time for three tactics.** The plots include a ribbon of width ± half the approximate LSD (5%) so that overlapping ribbons indicate that the predicted values are not significantly different.

The longitudinal data for total area from day 21 to day 51 is displayed in an additional figure (see Additional file 1). The mixed models fitted to this data showed that (i) the variance differed between Tactics (*P* = 0.004), (ii) of the possible random deviations from the trend over time, the significant deviations were those depending on the Tactic and Lane combination (*P* = 0.020) and on the Tactic and Position combination (*P* = 0.001), (iii) the trends over time are described by splines that vary between individual plants (*P* < 0.001) and with Tactic (*P* < 0.001), (iv) the trend over time did not vary between Lanes (*P* = 0.641 for the spline term and *P* = 0.419 for the linear term), and (v) there is a linear trend over Positions that varies with Tactic (*P* < 0.001), the splines over Positions being not significant (*P* = 0.210). There is correlation between the measurements on different days (*P* < 0.001), this correlation decreasing with the number of days between measurements and varying between Tactics. It is expressed as *ρ*^*d*^, where *ρ* is the correlation between measurements separated by one day and *d* is the number of days between measurements. The estimated values of the correlation coefficient for the Tactics are 0.422, 0.456 and 0.312, respectively. The fitted time trends for the different tactics are in Figure 3; the trend differs for all three tactics.

### Lane and position trends in the three multiline experiments

While the PA experiment is suited to examining east–west trends in the Smarthouse, lane trends cannot be investigated. Three two-phase multiline experiments were also conducted in 2011. Each filled the same Smarthouse as the PA experiment and so lane trends can be ascertained from them. Mixed models fitted to each of their results revealed that plant-to-plant variability is the main source of variation and this varied between the experiments, although the magnitude of this also differed between zones in some experiments. The CVs are in Table 5. Clearly, zones 3 and 4 in experiment 1 exhibited much greater variability than any of the other zones in any of the experiments, these being the more northern zones. In experiment 2, there was lower variance in zone 3, but the mean was also lower in that zone. The variances in the different zones of experiment 3 were not significantly different (*P* = 0.177); the differences in zone CVs are due to differences in the zone means.

**Table 5:** Coefficients of variation (%) in the different zones for the multiline experiments

	**Zone**^**a**^			
**Experiment**	**1**	**2**	**3**	**4**
1	20.06	18.70	32.13	76.82
2	17.81	15.49	16.38	
3	17.01	16.95	16.05	21.94

^a^Zones are numbered from the south to the north.

**Figure 4:**
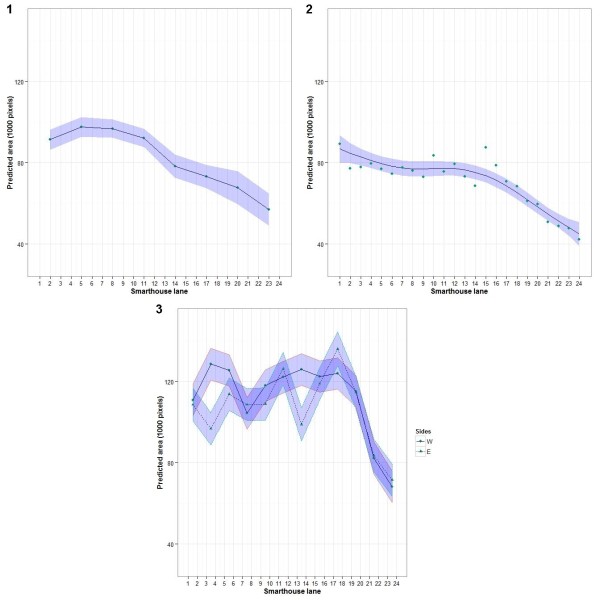
**Trend in total area across the lanes of the Smarthouse for the three multiline experiments. **The trend is based on predicted values for the random effects of (**1**) trios of lanes, (**2**) single lanes, and (**3**) pairs of lanes, respectively; a spline fitted the trend for just experiment (**2**) and it is shown; the trend varied randomly between the east and west sides only in experiment (**3**); the plots include a ribbon of width equal to the 95% confidence intervals and so the overlapping, or not, of ribbons does not indicate whether the predicted values are significantly different.

In all three multiline experiments there was a trend for lanes to decrease in total area from the south (lane 1) to the north (lane 24) as illustrated in Figure 4, although only in experiment 2 was the trend smooth. It appears that dividing the lanes into sets of four lanes would produce sets, each of which is homogeneous. The decrease in total area, which is focused on the northern end, is attributed to shading of some of the lanes at the northern end of the Smarthouse by the equipment in the adjoining imaging room (see Methods section). It was observed on 8 May 2012 that lanes 18–24 are shaded at midday. The extent of the decrease in total area was less for experiment 3. The plants were placed in the Smarthouse on 17 May 2011 for experiment 1, on 28 July 2011 for experiment 2, and on 12 September 2011 for experiment 3. The altitude of the sun was approximately 35° for the first two experiments and 50° for experiment 3 (Source: http://www.ga.gov.au/geodesy/astro/smpos.jsp, last accessed 11 April 2012). The greater height of the sun in September would have resulted in less shading from the imaging equipment and so explain the restriction of the decrease to the last four lanes of the Smarthouse. In the first experiment, there were significantly greater total areas for plants on the west side as compared to the east side of the Smarthouse. In the other two experiments, total area tended to increase towards the eastern side.

A summary of the relative efficiencies for detecting line difference in analyses of the multiline experiments that include lane or position trends, as compared to the analysis with no trends included, is given in Table 6. Clearly, gains in efficiency of 40% or more can be expected from allowing for lane trends. On the other hand, no more than a 10% gain in efficiency can be expected from allowing for position trends, and this is provided that lane trends have been allowed for. This is consistent with the results from the PA experiment.

**Table 6:** **Relative efficiencies**^**a**^**for line differences resulting from taking lane and position trends into account for the multiline experiments**

	**Experiment**		
**Trend terms in the analysis**	**1**	**2**	**3**
No trend	100.0	100.0	100.0
Lane trend	139.9	236.07	146.8
Position trend	102.8	95.9	98.0
Lane + Position trend	148.8	230.1	147.7

^a^The efficiencies are relative to the no-trend analysis.

### Comparison of alternative designs and analyses

Each tactic in the PA experiment can be viewed as a uniformity trial and so can be used to compare different designs for investigating treatments, for example a set of lines. Given that each tactic involves just three homogeneous lanes, our investigation of alternative designs using this experiment essentially considers only how best to block for east–west trends.

**Figure 5:**
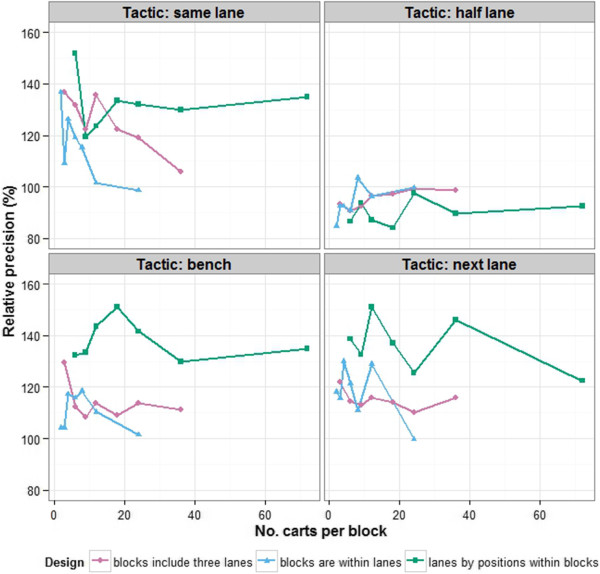
**Precision, relative to no blocking, of different blocking arrangements for each tactic. **Three series of blocking arrangements are considered: (i) blocks that are confined to the same lane of the Smarthouse with 2, 3, 4, 6, 8, 12 or 24 carts within a block, (ii) blocks that are contained within 1, 2, 3, 4, 6, 8, or 12 positions of the Smarthouse and spread across the 3 lanes so that they have 3, 6, 9, 12, 18, 24 or 36 carts within a block, and (iii) rows and columns in blocks each of 3 lanes by 2, 3, 4, 6, 8, 12 or 24 positions. Values greater than 100 indicate better precision than no blocking.

First, we focus on how blocks might be formed in the Smarthouse based on the total areas for day 51. Figure 5 gives the precision, relative to no blocking, of several potential blocking arrangements. It shows that precision for the half-lane tactic cannot be improved by blocking. For the other tactics there is a general tendency for the precision of the block designs to fall away as the block size increases, this being most pronounced for the same-lane tactic. For designs with rows and columns, the relative precision displays a flatter trend, with a few combinations showing superior precision. However, the designs with the better relative precisions vary between tactics. Of the arrangements that do not include rows and columns, blocks that have the following combinations of numbers of lanes and positions give better precision: 3 × 1, 3 × 2, 3 × 4 and 1 × 2 for the same-lane tactic; 1 × 4 and 1 × 12 for the next-lane tactic, although all the other blocking arrangements, except 1 × 24, are only a little less precise; 3 × 1 is the best blocking arrangements for the bench tactic. Designs with rows and columns within blocks of larger size have better precision than just blocks of a similar size; increased precision is likely to be obtained if 3 lanes by 6, 8, 12 or 24 positions are used.

**Figure 6:**
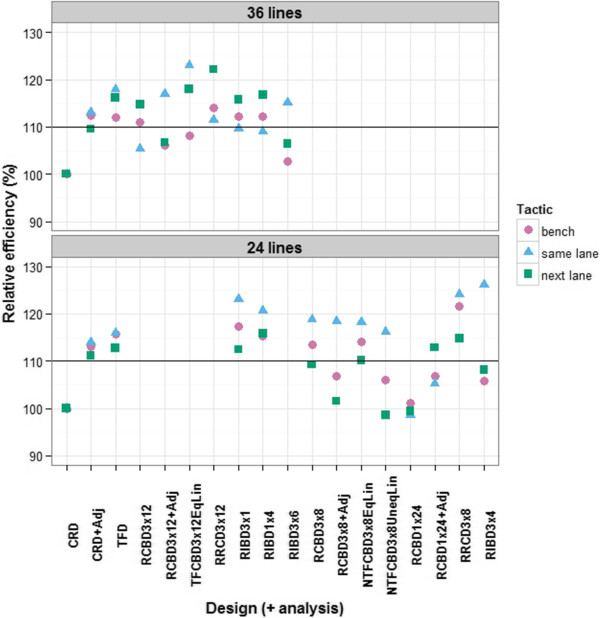
**Efficiencies, relative to a completely randomized design, of several designs for either 36 or 24 lines. **Lines are equally-replicated in their assignment to the 72 carts arranged in a grid of 3 lanes by 24 positions, for each of 3 PA tactics. A line parallel to the X-axis has been drawn at a relative efficiency of 110% to emphasize those situations in which an increase of at least 10% in efficiency can be expected.

However, relative precision is not the only consideration in comparing designs — other aspects, outlined in the Methods section, are also taken into account using relative efficiencies. So next, the relative efficiencies of designs and analyses selected for their better precision are examined, beginning with designs for 36 lines, all replicated twice. Reflecting on all the results so far, we investigate the relative efficiencies of the following designs and analyses: (i) a completely randomized design (CRD); (ii) a CRD with adjustment for trend across the positions (CRD+Adj); (iii) a trend-free design without blocking (TFD); (iv) a randomized complete block designs with two replicates of 3 lanes by 12 positions (RCBD3x12); (v) an RCBD3x12 with adjustment for trend across positions within a block (RCBD3x12+Adj); (vi) a trend-free design with two replicates of 3 lanes by 12 positions in which allowance is made for trends with equal slopes between blocks (TFCDB3x12EqLin), unequal slopes between blocks being inestimable; (vii) a resolved row-column design with 3 lanes by 12 positions in each replicate (RRCD3x12); (viii) a resolved incomplete block design with two replicates containing 12 blocks consisting of 3 lanes from the same position (RIBD3x1); (ix) a resolved incomplete block design with two replicates containing 9 blocks consisting of 4 consecutive positions from the same lane (RIBD1x4); and, (x) a resolved incomplete block design with two replicates containing 2 blocks consisting of 3 lanes by 6 columns (RIBD3x6). In all designs, except the CRD, a replicate consists of 3 lanes by 12 positions and contains one complete set of the treatments. The relative efficiencies (*RE*_PDA_) for these designs and analyses with 36 lines, when run on each of the bench, same-lane and next-lane tactics, are given in Figure 6. It is noted that all the values shown are based on Monte Carlo samples of the randomizations and, in the case of (nearly) trend-free designs, regenerations of the design; exact values can be computed for the RCBD3x12, but they differ by less than 1% from the simulated values. We conclude that in general the simulated values are likely to be accurate to ±1%.

The designs and analyses that are at least 10% more efficient than a CRD for all 3 tactics from the PA experiment are the CRD+Adj, the TFD, the RRCD3x12, the RIBD3x1, and the RIBD1x4. There is very little difference in efficiency between the RRCD3x12 and the RIBD3x1. The essential difference between the two designs is that, while both separate Position effects, the former isolates Lane differences as well, whereas the latter does not. Ignoring the bench tactic, the most efficient design and analysis is the TFCBD3x12EqLin, with TFD only slightly less efficient.

Relative efficiencies for a similar set of designs and analyses, but with 24 thrice-replicated lines, are also given in Figure 6. In this case, when there are blocks, only nearly trend-free designs (NTFD) are possible; however, in designs with blocks, they allow for unequal trend-slopes between blocks. In particular a nearly trend-free design with three replicates of 3 lanes by 8 positions is investigated, with equal slopes for the different blocks (NTFCBD3x8EqLin) and with different slopes for the different blocks (NTFCBD3x8UneqLin). The efficiencies show a similar pattern to that for 36 treatments, although the efficiencies are generally greater for the 24-line designs; the 24-line designs have more replication. In this case, none of the (nearly) trend-free designs perform better than the other designs and analyses; it seems that, with the smaller blocks, there is nothing to be gained from their use.

## Discussion

### Trends in the greenhouse and Smarthouse

It is concluded that, in the greenhouse, trends can occur down its length in even approximately 3 weeks of growth (Figure 2A). We believe that, in this case, the higher growth at the eastern end of this greenhouse, particularly on the southern side, was because these plants are next to an external eastern wall and so they received more light in the early morning. On the other hand, differences over the short distance encompassed by three rows of pots are unlikely, although there was evidence of a difference between sides.

The results of the analysis for the PA experiment (Figure 2) established that there is an east–west trend in the Smarthouse for the same-lane tactic, and that this has overshadowed minor column trends in the north-west of the greenhouse. One contributing factor to a Smarthouse position trend is the greater exposure of plants in the western half to the effects of the air conditioners. Presumably, the same is true for the bench tactic, although it cannot be confirmed because there is no data from day 21 for this tactic. For the half-lane tactic, the minor column trend in the north-east of the greenhouse has been also overshadowed in the Smarthouse, but in this case to produce no position trend for reasons discussed below. In contrast, the more pronounced column trend for total area in the south-east of the greenhouse is paralleled by a similar position trend in the day 51 total area for the next-lane tactic. The evidence for this is the disappearance of the position trend when the total area for day 51 is adjusted for the total area for day 21 using an analysis of covariance. It is not possible to be certain of the source of the position trend in total area from day 51. In particular, the contribution of the column trend in the greenhouse to it cannot be determined. On the other hand, it seems most likely that, like for the same-lane tactic, there is a contribution by the Smarthouse phase to the position trend in the day 51 total area for the next-lane tactic, although the trend might not be as great as in other tactics because of the suppressed growth for this tactic in the Smarthouse phase. Ultimately, the origin of the position trend is of little import here, because column trends in the greenhouse are aligned with position trends in the Smarthouse: whatever measures are taken to deal with one will deal with the other.

The three multiline experiments have shown that there is a trend for growth to decrease from south to north in the Smarthouse (Figure 4). This is in large part due to shading of some of the lanes at the northern end of the Smarthouse by the equipment in the adjoining imaging room, the number of shaded lanes being a maximum in winter. However, the PA experiment revealed that there were no differences within sets of three lanes in Smarthouse and the multiline experiments confirm this, although perhaps even sets of four lanes are homogeneous.

### Thigmomorphogenic or other movement effects

Predicted averages and variances generally differed between tactics in the PA experiment (Table 2). However, there was no average or variability difference between the bench and same-lane tactics for any of the responses from day 51, in particular, height or density index. Thus the movement three times a week for imaging and watering had no effect over and above that associated with traditional greenhouse practices. We infer from this that there was no thigmomorphogenic or other effects of movement in the Smarthouse. The lack of a thigmomorphogenic effect is perhaps not surprising given that no such effect has been found in wheat when the plants were stimulated by rubbing [5]. It would also appear that the potential effects of pot movement on the soil, and thence on plant growth, have been circumvented by the soil substrate chosen for use in this experiment (see the Methods section).

### Relocation of plants versus experimental design and statistical analysis

The results of the half-lane and next-lane tactics are informative in considering the issue of how to deal with microclimate variation: relocation of plants or experimental design and statistical analysis. At first sight, it may seem that relocation of plants is the better option because, as seen in the half-lane and next-lane tactics, trends can be reduced and perhaps nullified by appropriate movement. We now discuss why this may not be the case.

The half-lane tactic differed from the other tactics in displaying no east–west trend over positions (Figure 2B), because plants spent half their time in the each half of the Smarthouse. However, in order for plant relocation to be successful, it must result in plant variability that is similar to that for plants that maintain their position, as in the same-lane tactic, after lane and position trends have been removed in a statistical analysis of the data. This did not happen for the half-lane tactic; instead, while no east–west trend was detected, the variability of plants was inflated, relative to that for the other tactics. The magnitude of this inflation was similar to the amount of variation that is removed by a position trend in the bench and same-lane tactics. It is noted that, while plants have spent time in both the east and west halves of the Smarthouse, there are still differences between plants in the exposure to the east–west trend. Within a set of 12 plants that start in the same half, plants retain their east–west order for the whole experiment. Also, plants that start together in the middle of a lane spend half their time at opposite ends of the lane. That is, while the half-lane tactic does reduce the trend to the extent that it was not detectable, it does not eliminate it because plants are not equalized with respect to the trend. Further, the tactic increases the inequality in exposure.

The next-lane plants, compared to same-lane plants, have smaller total area on day 51, are less variable for total area on day 51, are significantly shorter on average and have a lower density index (Table 2). This is consistent with the next-lane plants having been shaded during their growth. Further, evidence for this shading effect comes from the three multiline experiments, for which we argue that some of the lanes at the northern end of the Smarthouse are shaded by the equipment in the adjoining imaging room. At the time of the year that the PA experiment was run, it would have been the 6 most northern lanes at most that were shaded during it. That is, the plants in the next-lane tactic would have been shaded during only part of their time in the Smarthouse. They would have entered the shaded area sometime after the 6^th^ time point, depending on how many lanes were shaded. However, all would have been shaded for the same amount of time, the number of time points spent in the shade being equal to the number of shaded lanes. The first lane of the tactic would have entered and left the shaded area two time points after the third lane, which is 5 days or less. So, any retardation in growth would begin after at least the 6^th^ time point (day 32) and this is what is observed in Figure 3. There is also evidence of an increased growth rate after time point 12, time point 13 being the point at which all lanes have emerged from the shade. The lack of a difference between the three lanes for the next-lane tactic confirms that the effect of shading during the Smarthouse phase was similar for all the plants in this tactic. The variance of plants in this tactic was smaller than for the same-lane or bench tactics. A smaller variance for this zone was also observed in multiline experiments 2 and 3, in which carts were always returned to the same position. This suggests that the smaller variance for plants in the next-lane tactic is most likely due to the reduced growth of plants in this tactic, rather than the more equal exposure of plants to the microclimates in the Smarthouse leading to reduced variability. In any case, this decrease in variance would only be beneficial in an experiment involving multiple lines if there was not a matching reduction in the differences between lines.

Clearly, both half-lane and next-lane tactics have had the effect of spreading microclimate effects across all the plants in these tactics, position trends in the first case and lane trends in the other. However, while the half-lane tactic does not equalize the plants experience of the east–west trend, the next-lane tactic evens out the exposure of the plants to the north–south trend. This demonstrates that for rearrangement of plants during the experiment to be an effective strategy requires that the plants experience equally every microclimate in the experimental area. Even if this is achieved, the precision of the experiment will be no better than can be achieved by adjusting for trends in the analysis. The reason for this is that the effect of rearrangement is limited to removing microclimate differences, such as can be adjusted for in the statistical analysis, but has no effect on the other sources of variation in the experiment, such a soil and plant variability.

Attaining equal exposure to microclimates is probably easiest with systematic relocation, such as was used with the half-lane and next-lane tactics. Even so, while accomplishing equalization in small experiments may well be practicable, it is likely to be difficult to achieve in large experiments. For example, consider an experiment to be conducted in a Smarthouse that occupies 24 lanes by 24 positions. We have identified that areas of 4 lanes by 6 positions are reasonably homogeneous in our Smarthouse, which means that in the proposed experiment there are 6 by 4 or 24 such areas. The relocation strategy would need to rearrange the plants in the experiment so that each of 24 groups of 24 plants is located for the same amount of time in each of these 24 areas. This is not possible in a 31 day experiment. It would be for a 24 day experiment, but then, for each area, some plants would start the experiment in that area and other plants would finish in it; these plants would be at different stages in their growth. On the assumption of the same east–west trend for all lanes, it would only be necessary to ensure that plants spent the same amount of time in each of the 4 sets of 6 positions and the 6 sets of 4 lanes. This could be done in 12 days. Our data support such an assumption.

On the other hand, random or haphazard relocation of plant during an experiment will not equalize plant exposure to microclimates. Rather it will make it difficult, if not impossible, to adjust for microclimate differences and so will almost certainly result in greater variance than if adjustment can be made.

### Which experimental design and statistical analysis?

Given that microclimate differences are to be accounted for by experimental design and statistical analysis, rather than relocation of the plant during an experiment, the question that arises is which experimental designs and statistical analysis are best as far as minimizing the variance of treatment differences is concerned. In answering this question, our investigation of alternative designs using total area from the PA experiment is relevant to dealing with the east–west trend, while the three multiline experiments provide information about the north–south trend. The result of these investigations (Figures 4, 5 and 6) is that, in general, blocks should be as small as possible, consisting of 4 lanes by 4 or 6 positions. It might appear that small blocks are the obvious solution, but this is not necessarily the case. While one would expect smaller blocks to be more homogeneous, and so be preferred, there are other elements of an experiment that may result in greater efficiency for larger blocks. In particular, with larger blocks, the amount of information estimated from within blocks will be higher and the error variance will be more precisely estimated, thereby counterbalancing the superior homogeneity of smaller blocks. Our results show that alternatives to small blocks are to use (nearly) trend-free designs with larger blocks and fit position trends as equal slopes for blocks or, as a last resort, blocks of several complete rows with trend terms for position in the analysis.

In the PA experiment, the exposure of plants to the increasing trend in total area from west to east in the greenhouse was aligned with their exposure to a trend from west to east in the Smarthouse. Consequently, the PA experiment conforms to Principle 8 (Big with big) in [18] in that comparisons between greenhouse columns and between Smarthouse positions are confounded with each other. This means that whatever steps are taken to adjust for east–west trend will do so simultaneously for the greenhouse and the Smarthouse. It also has the advantage of keeping the design simple and so observing Principle 5 (Simplicity desirable) in [18].

### How many replicates?

An important issue in designing an experiment is the number of replicates for each treatment. Unfortunately it is impractical to give general guidelines because the number of replicates for each response variable depends on the amount of variation to be expected, the size of the difference to be detected, the number of treatment to be employed, how the error degrees of freedom are calculated, the significance levels to be used and the power required. Many different combinations of the values for these quantities occur, even in greenhouse experiments, and so the number of replicates will vary between experiments. The contribution of this paper is in suggesting ways in which the amount of variation to be expected can be minimized. Further, the results in this paper suggest that a CV in the range 20% to 30% can be expected in total area for day 51 in such experiments (see Tables 2 and 5). If one expresses the difference to be detected as a percentage of the expected mean value, then this value can be used in calculating the number of replicates required.

### A limitation

A limitation of the PA experiment is that each tactic was applied in only one zone, this being a necessary, practical restriction. We are of the opinion that this is unlikely to have affected our comparison of the bench and same-lane tactics, these being located next to each other and covering no more than 6 lanes at the unshaded, southern end of the room. Our main results for the half-lane and next-lane tactics are concerned with the position trend. It would appear that the position trend is consistent across the whole Smarthouse as the slope does not differ significantly between the bench, same-lane and next-lane tactics. However, while we also consider it unlikely, we cannot rule out that the extra variability associated with the half-lane tactic is due to its being in a zone that is inherently more variable than the other zones.

## Conclusions

The movement three times a week for imaging and watering in the Smarthouse had no thigmomorphogenic or other effect, over and above that associated with traditional greenhouse practices.

It is concluded that the decrease in variability arising from relocation in a greenhouse, hoped for in [3], will occur provided the plants are equalized in their experience of the microclimates present in the experiment. However, an appropriate experimental design and analysis will achieve the same result more easily and reliably and so is to be preferred.

The results of the PA and the multiline experiments indicate that spatial designs are not required in greenhouse experiments involving single-plant pots on a conveyor system. Further, they suggest that complete or incomplete block designs or, when blocks are larger, (nearly) trend-free designs may well be better suited to such greenhouse experiments than designs with rows and columns. Of course, it will depend on the configuration of the greenhouse and Smarthouse. In general, to take account of variation in microclimate in a particular greenhouse, the options are: (i) blocking in designing and analysing an experiment, (ii) the inclusion of trend terms in the analysis or, (iii) when blocks are larger, a (nearly) trend-free design. Experiments using one of these options are likely to be more efficient than those in which the positions of plants are rearranged during the experiment.

In our case, any blocking in the greenhouse should ensure that pots close to external walls are in different blocks to other pots. In our Smarthouse, blocks need to account for the substantial north–south trend and the smaller east–west trend as well. The results of our investigation indicate that there is little difference over 3 or 4 lanes and so it is advantageous to form blocks consisting of up to 4 lanes. There is a smaller east–west trend, but the use of appropriate designs and analysis has been shown to produce at least a 10% increase in efficiency. Overall, it has been demonstrated that more than a 40% increase in efficiency can be achieved, with blocking of lanes being the most important contributor to this. It should be noted that this conclusion applies to total area and similarly-behaved measurements. It would not be valid for a variable that changes substantially during the day and so would change during the hour or so that it takes to measure 3 or 4 lanes. Such a variable would need to have blocks within lanes.

Being a two-phase experiment, the principles outlined in [18] are relevant. Here we recommend that the arrangement in the greenhouse parallel that in the Smarthouse, but with randomization where appropriate. The general principle is that sources of significant variation in the greenhouse should be associated with such sources in the Smarthouse, thus satisfying Principle 8 (Big with big) in [18]. For example, blocks in the greenhouse randomized to blocks in the Smarthouse and trend in the greenhouse associated with trend in the Smarthouse.

We acknowledge that the greenhouse facility employed in the PA experiment we report, The Plant Accelerator^®^, is not typical of those in use more broadly and so our conclusions are not necessarily applicable to standard greenhouses. However, in our experience, the behaviour that we have observed in the PA experiment is similar to that which occurs in greenhouse experiments more broadly. We expect that our general conclusions will apply, but that their specific application to other situations requires investigation of the local circumstances.

## Methods

### The PA experiment

The experiment used seed from a single line of wheat (*Triticum aestivum*), Gladius (AGT), and it is a two-phase experiment. Seed was planted in a greenhouse on 6 June 2011 and the plants moved to a Smarthouse on 24 June 2011. They remained in the Smarthouse until the 27 July 2011, when they were harvested and the shoot fresh weight measured. Seeds were obtained directly from Australian Grain Technologies (AGT) and three seeds were planted in each pot. The soil substrate used was specifically designed for the use on a conveyor system, consisting of about 50% (v/v) sand, 35% (v/v) coco-peat and 15% (v/v) clay/loam with minerals and slow release fertilizer added (Osmocote Exact Mini 16+3+9+1.2Mg+TE). The substrate has a high enough sand content to reduce compaction on the belt and at the same time reduces soil shifting within the pot due to the peat and clay content. After germination only one plant was retained in each pot, plants being selected so that those remaining were as similar as possible. While in the Smarthouse, each plant was imaged three times a week, on Mondays, Wednesdays and Fridays, resulting in each plant being imaged on each of 14 days; they were weighed and watered twice a week, on Mondays and Fridays, immediately after imaging. The imaging of a plant involved taking three 5 megapixel RGB images: one top view image and two side view images at a 90° horizontal rotation. These images were processed to obtain the area of plant exhibited in each image and the total area calculated by summing the areas for the three images. The total area calculated in this way has been shown to be related to the shoot dry weight of the plant [17]. The height of the plant was also obtained from the two horizontal images and their maximum taken as a measure of the height, dividing by 19.5 to convert the measurement to centimetres. A density index for the plant was obtained as the ratio of the total area to the height. One would expect thinner plants to have smaller values of this index.

**Figure 7:**
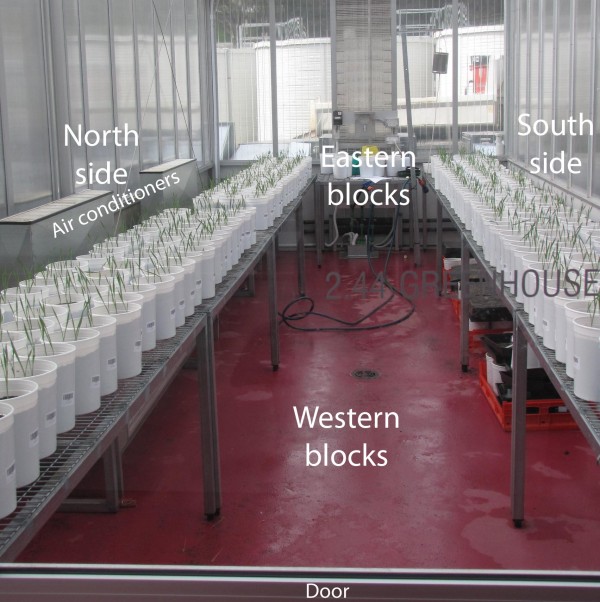
The layout of the plants in the greenhouse.

The greenhouse in the first phase was aligned on a west/east axis with a door at the western end and two air conditioners on the northern wall. The layout of the plants in this greenhouse is shown in Figure 7. There was a row of tables on each of the northern and southern sides of the door and on each side there was an eastern and a western block. Thus there were 4 locations and each of these contained a grid of 3 rows by 24 columns of pots.

**Figure 8:**
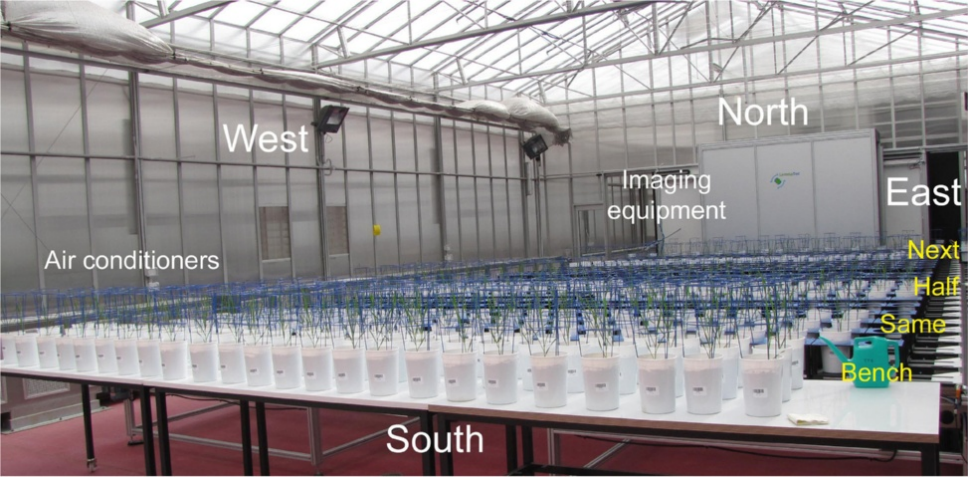
The layout of the plants in the Smarthouse.

The Smarthouse conveyor system has capacity for 624 pots arranged in 24 Lanes of 26 carts, with lanes numbered from south to north and positions of carts within a lane from west to east. There are air conditioners along the western side and the imaging equipment is located in an adjoining room to the north of the Smarthouse (Figure 8). For the PA experiment, only 24 carts per lane were used and the Smarthouse was divided into four zones: (i) a set of benches, (ii) lanes 1–3, (iii) lanes 4–11, and (iv) lanes 12–24. The pots from a location in the greenhouse were placed initially in a grid of 3 lanes by 24 positions in the Smarthouse, the order of plants within a block from the greenhouse being maintained in the Smarthouse. That is, there is a direct correspondence between the Row-Column coordinates in the greenhouse and the Lane-Position coordinates in the Smarthouse. However, the location to be placed in a zone was randomly selected. In this phase, one of four tactics outlined in the Background section was used with the plants in a zone. The layout of the plants in the Smarthouse is shown in Figure 8.

The factor allocation for the PA experiment is summarized in the factor-allocation diagram in Figure 1B. The allocations shown are coincident [19] in that, in the allocations of pots and treatments to carts, both Tactics and the four combinations of Sides and Blocks are assigned to the Zones.

The design used will allow the assessment of combined east/west trends across columns and positions. It is possible to determine if trends become established across the columns in the greenhouse, through the analysis of day 21 observations when it can be assumed that any influence of the Smarthouse will be negligible. However, it is not possible to separate the contributions of greenhouse and Smarthouse to any position trends that are observed in the total areas for day 51. North/south trends across sides and rows in the greenhouse can also be evaluated using day 21 observations. However, north/south trends across lanes in the Smarthouse are not be fully assessable in this experiment.

### Three multiline experiments

**Figure 9:**
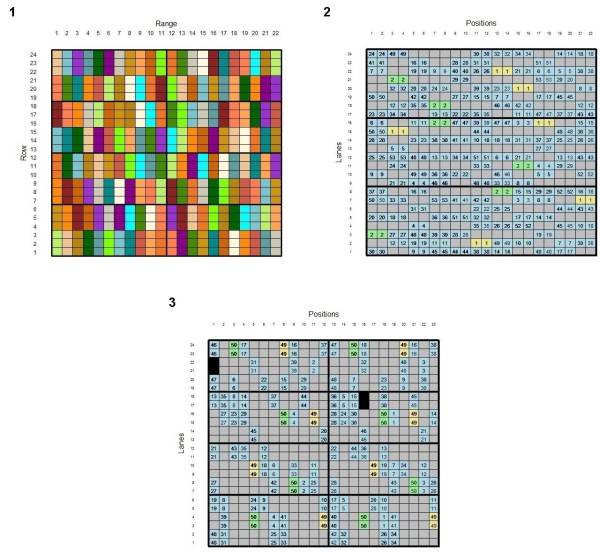
**The arrangements of lines in the Smarthouse for the three multiline experiments. **Each cell in a diagram represents a subplot. In experiment 1, each colour corresponds to a line and adjacent subplots have the same colour. In experiments 2 and 3, blue corresponds to a line that is replicated twice, grey to a line that is unreplicated and green and yellow to the two parent lines; subplots for the same line have the same number, except for the unreplicated lines for which the subplots form main plots in the same manner as for the other lines. Blocks are indicated by thick black lines.

Three multiline experiments conducted in The Plant Accelerator^®^ investigated lines of wheat grown under different conditions. Each of them took up the whole of the same Smarthouse as the PA experiment. All of them employed split-plot designs, with the lines assigned to the main plots and the conditions randomized to the subplots (carts); each main plot consisted of 2 or 3 carts depending on the experiment. The arrangements of the lines in the Smarthouse for these experiments are illustrated in Figure 9. The experimental designs used for the three experiments are summarized as follows:

1. 22 varieties were grown under 3 conditions using 24 lanes by 22 positions; the 24 lanes were divided into 4 zones each of 6 lanes and the 22 positions into 2 sides each of 11 positions; the combinations of the 4 zones with the 2 sides formed 8 blocks that contained a replicate of the varieties-conditions combinations; within each block the 6 lanes were divided into 2 strips of 3 lanes; a main plot consisted of the 3 adjacent carts from a strip in one of the positions; a complete set of the varieties were assigned to the main plots in each block using an equally-replicated spatial design generated with the R [20] package DiGGer [21]; the 3 conditions were randomized to carts within a main plot; the plants to go in each block of 6 lanes by 11 positions were placed in the greenhouse together, sometimes in 6 rows by 11 columns, but in other cases in irregular configurations; the 3 pots for a main plot were usually adjacent.

2. 153 lines were grown under 2 conditions using 24 lanes by 22 positions; the lines were applied to main plots using a partially-replicated spatial design [13] generated with the R [20] package DiGGer [21]; in this design the experimental area was divided into 3 zones of 8 lanes, within each of which the main plots were arranged in 8 lanes by 11 pairs; the 2 conditions were randomized to pairs of consecutive carts in the same lane; the plants to go in each block of 8 lanes by 22 positions were placed in the greenhouse in 16 rows by 11 columns, with the 2 pots for a main plot in adjacent rows.

3. 214 lines were grown under 2 conditions using 24 lanes by 23 positions; the lines were applied to main plots using an augmented block design; as for the first experiment, the experimental area was divided into 8 blocks arranged in a rectangle of 4 zones by 2 sides; within each block, the main plots were arranged in 3 strips by 12 positions on the left side of the rectangle and 3 strips by 11 positions on the right side of the rectangle; a strip of main plots consists of 2 lanes and the 2 conditions were randomized to carts in 2 adjacent lanes in the same position; the plants to go in each block of 6 lanes by 12 or 11 positions were placed in the greenhouse in 6 rows by 12 or 11 columns.

These experiments are included in this paper in order to investigate the trends in the Smarthouse. This will be done by estimating the effects for strips of main plots and the effects of positions. It is noted that, given the results of this paper, we would no longer use spatial designs for experiments like this.

### Statistical analyses

The response variables for the PA experiment, whose analysis we report, are the total areas on day 21, the first day of plant imaging, for all but the plants going to the benches in the Smarthouse, and the fresh weights, total areas, height and density index on day 51, the harvest date. We first plotted row profiles of the raw data in order to gain an impression of the responses (see Additional file 1). To assess the sources of variation active in the PA experiment, mixed models were fitted using GenStat ([22], Chapter 5) that uses the numerical routines from the standalone program ASReml™ [23]. The models were formulated as described in [24] and we express them using the notation in ([24], Table 1). A term in a model consists of a set of one or more factors, with multiple factors separated by a ‘wedge’ (‘∧’) that indicates the term is for the combinations of the levels of those factors. For example, the term Side∧Row is a term for all 6 rows, 3 from each side. The model is formed as the sum of two sets of terms, the sum to the left of a ‘straight line’ (‘|’) are considered fixed while those to the right are considered random. However, the mechanics of the fitting is that spline terms are fitted and tested as part of the random model even though they model systematic behaviour in the data and so are shown as fixed terms.

The sequence of fitting, following that described in [24], is:

1. An analysis-of-variance model is fit that does not include any trend terms, in which variances for all terms are homogeneous and in which there is no autocorrelation between plants. In this model, all terms are fixed except the residual error term, the term that involves all factors.

2. A term is added that allows unequal residual variances between Locations or Tactics, these being the most likely source of heterogeneous variance, given the physical layout of the experiment.

3. Having decided to reject or retain unequal variances, we include autocorrelation between Columns or Positions that is allowed to differ between Locations or Tactics; autocorrelation between Rows or Lanes is not appropriate as there are too few of them to estimate it. Inclusion and testing of this term allows an assessment of whether local spatial variation is present in the experiment, because there is a tendency for neighbouring plants to be similar.

4. Next, in order to examine global variation, in the form of east–west trends, the terms involving Columns or Positions are reparameterized to allow for systematic trends across their levels. Linear tends are fitted, as are curved trends, the latter being fitted using cubic smoothing splines [25]. The reparameterization consists of replacing each term (for example, Columns∧Blocks) by three terms: linear, spline and random deviations terms. For the first two terms, the factor Columns or Positions is placed in parentheses and preceded by ‘lin’ or ‘spl’; for the last term, the term is plain and designated as a constrained random term. Thus, to choose which of the following models best describes the trend associated with a term, hypothesis tests are performed in the following order, dropping nonsignificant random terms and stopping when a significant term is encountered: (i) there is no smooth trend because of significant random deviations, (ii) the trend is curved as evidenced by a significant spline term, the spline term being constrained to be nonnegative, (iii) the trend is linear as it has a significant linear term, or (iv) there is no effect associated with the particular set of factors because no terms involving that set are significant.

5. Then hypothesis tests for unequal residual variances between Locations or Tactics and autocorrelation between Columns or Positions are performed again to check whether these terms are needed in the context of the model chosen in the previous step.

6. Hypothesis tests for the nontrend, fixed terms were conducted. A test for a fixed term was not conducted if (i) its factors are a subset of those for a significant fixed term or (ii) it has the same factors as a higher-order fixed term that is significant. In the present context, random deviations terms are of higher order than spline terms, which are of higher order than linear terms. Nonsignificant fixed terms were not removed from the model.

Except where stated otherwise, all hypothesis tests employ a significance level of 0.05. However, we do not religiously omit terms with a *P*-value greater than 0.05; on occasion terms with a *P*-value between 0.05 and 0.10 are retained on the grounds that this is some indication that the terms are required. To test for terms in the random part of a fitted model Restricted Maximum Likelihood Ratio Tests (REMLRT) were used, the calculation of the *P*-value being adjusted when the test involved a variance component constrained to be nonnegative [25]. Tests for fixed effects were carried out using F-tests with Kenward-Roger adjustments [26]. The estimated denominator degrees of freedom for these tests were in excess of 125 for day 21 measurements and 140 for day 51 measurements. The standard errors of predicted averages are based on approximately 68 degrees of freedom when separate variances are estimated for each tactic and 272 degrees of freedom otherwise.

The analysis-of-variance model for measurements on day 21 is based on the factors in the leftmost panel of Figure 1B, because none of the other factors could have come into effect at this juncture. The model, with Locations substituted for Blocks∧Sides, is:

$$\begin{array}{l} \text{Locations}+\text{Locations}\wedge \text{Rows}+\text{Columns}\wedge \text{Locations}\\ \;|\text{Locations}\wedge \text{Rows}\wedge \text{Columns} \end{array}$$

The maximal model for measurements on day 21 includes the reparameterization of terms with Columns into three trend terms, heterogeneous variances for Locations and autocorrelation between Columns. The ‘idh’ preceding the Locations factor indicates that allowance is made in the model for unequal variance between the Locations. Similarly, ‘ar1’ is used to specify autocorrelation of order 1 between the levels of the columns factor. The maximal model is

$$\begin{array}{l} \text{Locations}+\text{Locations}\wedge \text{Rows}\;\\ \;+\text{lin}\left(\text{Columns}\right)\wedge \text{Locations}\\ \;+\text{spl}\left(\text{Columns}\right)\wedge \text{Locations}\\ \;|\text{Columns}\wedge \text{Locations}\\ \;+\text{idh}\left(\text{Locations}\right)\wedge \text{Rows}\wedge \mathrm{ar}1\left(\text{Columns}\right). \end{array}$$

On the other hand, the analysis-of-variance model for measurements on day 51 is based on the factors from the two righthand panels of Figure 1B; the factors in the leftmost panel are not included as they are equivalent to those in the middle panel and Zones is not included as it is equivalent to Tactics. The model is:

$$\begin{array}{l} \text{Tactics}+\text{Tactics}\wedge \text{Lanes}+\text{Positions}\\ \;+\text{Positions}\wedge \text{Tactics}|\text{Tactics}\wedge \text{Lanes}\wedge \text{Positions}\text{.} \end{array}$$

The maximal model is:

$$\begin{array}{l} \text{Tactics}+\text{Tactics}\wedge \text{Lanes}\\ \;+\text{lin}\left(\text{Positions}\right)+\text{lin}\left(\text{Positions}\right)\wedge \text{Tactics}+\text{spl}\left(\text{Positions}\right)+\text{spl}\left(\text{Positions}\right)\wedge \text{Tactics}\\ \;|\text{Positions}+\text{Positions}\wedge \text{Tactics}\\ \;+\text{idh}\left(\text{Tactics}\right)\wedge \text{Lanes}\wedge \mathrm{ar}1\left(\text{Positions}\right). \end{array}$$

In the above models, all terms except for the first and last, represent some form of global variation. The autocorrelation terms represent local spatial variation.

In addition, the extent to which the differences between the plants arising in the greenhouse phase are related to the total areas on day 51 are examined by including the total area on day 21 as a covariate in an analysis of total area on day 51. For this analysis of covariance, the bench tactic had to be omitted. The model for it began with the selected model for total area on day 51 to which were added linear and spline terms for total area on day 21 to allow for curvature, as well as terms allowing the relationship to differ between tactics. The analysis will adjust total areas on day 51 for differences in total areas on day 21.

Mixed models were also investigated for the longitudinal data for total area from day 21 to day 51 using GenStat ([22], Chapter 5) and ASReml-R [27], the latter being a package for the R statistical system [20] that uses the numerical routines from the standalone program ASReml™ [23]. These models took into account the results of the analyses for total area for days 21 and 51 and, in addition, included (i) trends over time that varied with tactic, (ii) random deviations from these trends, (iii) random deviation of a plant from the trend over time for its tactic, and (iv) correlation between time points that decreased as the distance between the time points increased, as indicated by the ‘exp’ function on Day.

The maximal mixed model was:

$$\begin{array}{l} \text{Tactic}+\text{Tactic}\wedge \text{Lane}+\text{lin}\left(\text{Position}\right)\wedge \text{Tactic}\\ \;+\text{spl}\left(\text{Position}\right)\wedge \text{Tactic}\\ \;+\text{lin}\left(\text{Day}\right)+\text{lin}\left(\text{Day}\right)\wedge \text{Tactic}\\ \;+\text{lin}\left(\text{Day}\right)\wedge \text{Tactic}\wedge \text{Lane}\\ \;+\text{spl}\left(\text{Day}\right)+\text{spl}\left(\text{Day}\right)\wedge \text{Tactic}\\ \;+\text{spl}\left(\text{Day}\right)\wedge \text{Tactic}\wedge \text{Lane}\;\\ \;|\text{Tactic}\wedge \text{Position}+\text{Tactic}\wedge \text{Position}\wedge \text{Lane}\\ \;+\text{Tactic}\wedge \text{Position}\wedge \text{Day}\\ \;+\text{Day}+\text{Day}\wedge \text{Tactic}+\text{Day}\wedge \text{Tactic}\wedge \text{Lane}\\ \;+\text{idh}\left(\text{Tactic}\right)\wedge \text{Position}\wedge \text{Lane})\wedge \text{spl}\left(\text{Day}\right)\\ \;+\text{idh}\left(\text{Tactic}\right)\wedge \text{Position}\wedge \text{Lane})\wedge \text{exp}\left(\text{Day}\right). \end{array}$$

Model fitting in this case began with all terms in the maximal model except the last. The fitting strategy used was that described in [24] in that, after testing for heterogeneous variances between Tactics, random trend modelling was followed by investigation of the covariance structure and finally testing of fixed terms was performed.

For the three multiline experiments, the only response variable analysed was the total area at the end of the Smarthouse phase. The analyses also involved fitting mixed models, but using ASReml-R [27], in a similar manner to that described for the PA experiment. The main differences are that unequal zone variances were incorporated into the model and tests performed to see if the model was significantly different to one with equal zone variances and trends across the lanes were fitted. In the case of the trends, they were fitted to the strips that consisted of three, one and two lanes in the three experiments, respectively. This is because differences between lanes within a strip are confounded with Conditions.

### Investigation of alternative designs

We first investigate the blocking that will result in the best precision (lowest error variance) for total area on day 51 in each zone in the Smarthouse phase, irrespective of any treatments that might be applied. While different zones were subject to different tactics, it is not inconceivable that similar plant behaviour to that in the different zones, except perhaps for that having the half-lane tactic, will occur in other experiments. For example, the bench tactic would be relevant for an experiment involving manual imaging and the next lane in situations where the whole of the experimental area is shaded. In any case, it will help to make the selected designs robust to a range of situations. To examine the relative merits of various alternative blocking arrangements, they are applied to each zone and the results analysed for each arrangement for each zone. That is, the null mixed model analysis, that ignores any treatments that might be applied, is obtained. Designs are compared using the relative precision, being the ratio of the error variance for an arrangement with no blocking to that for a proposed arrangement. The error variance for no blocking is the variance of the 72 observations for a zone. Designs with a relative precision greater than one will usually be preferred, although designs with larger block size have the advantage of greater error degrees of freedom.

Having identified appropriate blocking arrangements, these will be examined in more detail for specified treatment factors with particular numbers of levels. It is common for experiments run in The Plant Accelerator^®^ to have a large number of lines and few replicates. Hence, given 72 carts in a zone, experiments with 36 or 24 lines, each replicated twice or thrice, would be analogous to such experiments. Additionally, analyses in which adjustment is made for position trend are compared with those in which they are not, as are designs that are trend-free or nearly trend-free [28] compared with those that are not. The (nearly) trend-free designs arrange the treatments so that they are either orthogonal to (trend-free) or as close as possible to orthogonal to (nearly trend-free) trends, in this case, position trend. Then appropriate linear trend terms are included in the analysis to remove the effect of the trend. DiGGer [21], a package that runs in the R statistical system [20], is used to generate the designs. These designs have restricted randomizations. In general, the order can be reversed across the whole set of positions and the rows can be randomized. Additionally, if there are blocks, the block orders can also be permuted between blocks. Further, regeneration of a design in DiGGer results in different designs that are not merely the result of swapping treatment labels, which introduces a further random element to the design.

To compare designs, the relative efficiency of a completely randomized design to a proposed design or analysis, *RE*_PDA_, is used. It is based on the A-optimality criterion and, in effect, compares the average sizes of the confidence intervals for pairwise differences between predictions for treatments. Designs with smaller confidence intervals have greater efficiency. It is computed as follows:

$$RE_{\text{PDA}}=\frac{1/AP_{\text{PDA}}}{1/AP_{\text{CRD}}}=\frac{AP_{\text{CRD}}}{AP_{\text{PDA}}}$$

where *AP*_CRD_ and *AP*_PDA_ are the modified A-optimality criterion of [29] for a completely randomized design (CRD) and a proposed design or analysis (PDA), respectively. The value of AP for a design is calculated as $\mathrm{AP}=F_{1,d,1-\alpha }\bar{\sigma_{\text{diff}}^{2}}$ where *F*_1, *d*,1−*α*_ is the 1 − *α* quantile of the *F* distribution for 1 numerator and *d* denominator degrees of freedom, *d* is the error degrees of freedom and $\bar{\sigma_{\text{diff}}^{2}}$ is the average of the variances for all pairwise differences between the predictions for the combinations of a set of factors of interest in an experiment. In addition to the precision of the design, discussed above, this measure of efficiency depends upon the number of replicates for the treatments, the manner in which treatment information is confounded with the several sources of random variation in an experiment and the degrees of freedom associated with the error variance estimate on which $\bar{\sigma_{\text{diff}}^{2}}$ is based. This last aspect is accounted for with the inclusion of *F*_1, *d*,1−*α*_ into the criterion. In the case of orthogonal analyses, such as for completely randomized and randomized complete block designs without trend isolation, AP can be calculated using standard formulae for the standard error of pairwise differences in treatment means. In the other cases, it is approximated by (i) obtaining a Monte Carlo sample of size 5000 of the randomizations for the design (1000 for (nearly) trend-free designs), (ii) calculating, from a mixed model analysis for each randomization, the average of the variances for all pairwise differences between a set of predictions and (iii) taking the mean of these averages over all randomizations in the sample. Being mixed model analyses, the predictions are the result of combining information from all random sources of variation. The mean of the denominator degrees of freedom from the Monte Carlo sample and *α* = 0.05 is used for obtaining *F*_1, *d*,1 − *α*_.

## Competing interests

The authors declare that they have no competing interests.

## Authors’ contributions

CB and MT conceived the study. All authors were involved in planning the study. BB implemented the PA experiment. HR designed the multiline experiments. CB and HR performed the statistical analyses. CB wrote the first draft of the manuscript. All authors contributed to the revision of the manuscript and have given final approval of the version to be published

## Supplementary Material

Additional file 1 **Plots of observed data. **Two set of plots of the original raw data are presented: 1) row profiles for the measured responses on days 21 and 51 that show the column and position trends in the raw data; 2) trend in total area over time for individual plants. Click here for file
