# Supplementary material for: Accounting for variation in designing greenhouse experiments with special reference to greenhouses containing plants on conveyor systems
Source: Plant Methods. 2013 Feb 8;9:5. doi: 10.1186/1746-4811-9-5 (PMC3630016; doi:10.1186/1746-4811-9-5)
Supplement: Additional file 1 — Plots of observed data. Two set of plots of the original raw data are presented: 1) row profiles for the measured responses on days 21 and 51 that show the column and position trends in the raw data; 2) trend in total area over time for individual plants. [file 1746-4811-9-5-S1.pdf]

Plots of observed data

Row profiles for day 21 and 51

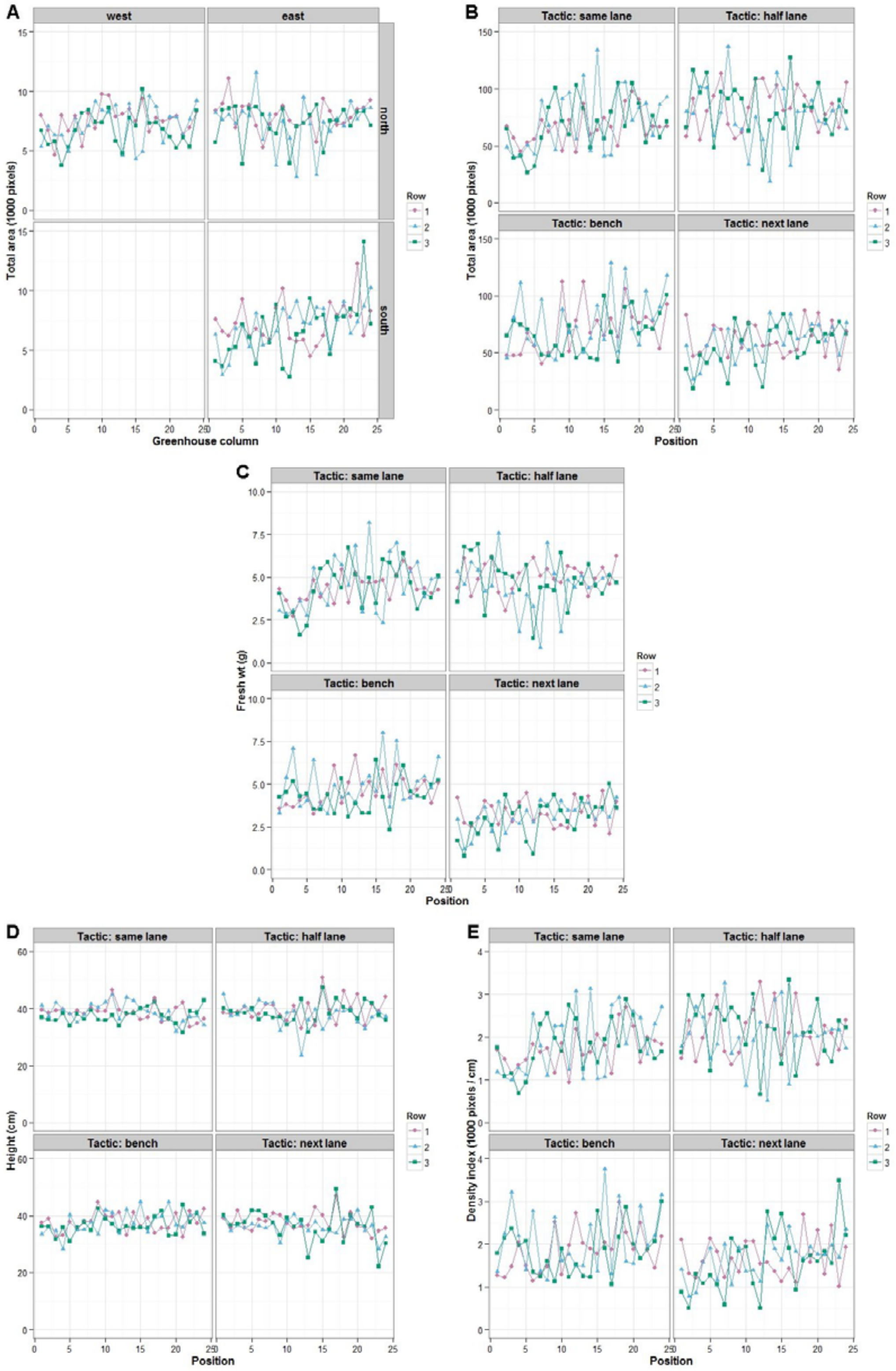

Figure 1 Row profiles of (A) total areas on day 21 versus greenhouse column for each location (B) total areas on day 51 versus Smarthouse position for each tactic, (C) fresh weights on day 51 versus Smarthouse position for each tactic, (D) height in day 51 versus Smarthouse position for each tactic and (E) density index on day 51 versus Smarthouse position for each tactic (each line in (A) corresponds to a greenhouse row and in the others corresponds to a Smarthouse lane). The graphs for the same response variable are arranged according to their geographic location in the greenhouse. In all graphs, considerable

plant-to-plant variability is exhibited. In several there is a tendency to increase from west (left) to east (right); for example, all same lane plots show this. In all plots on day 51, next lane has smaller values than the other tactics.

Trend in total area over time for individual plants

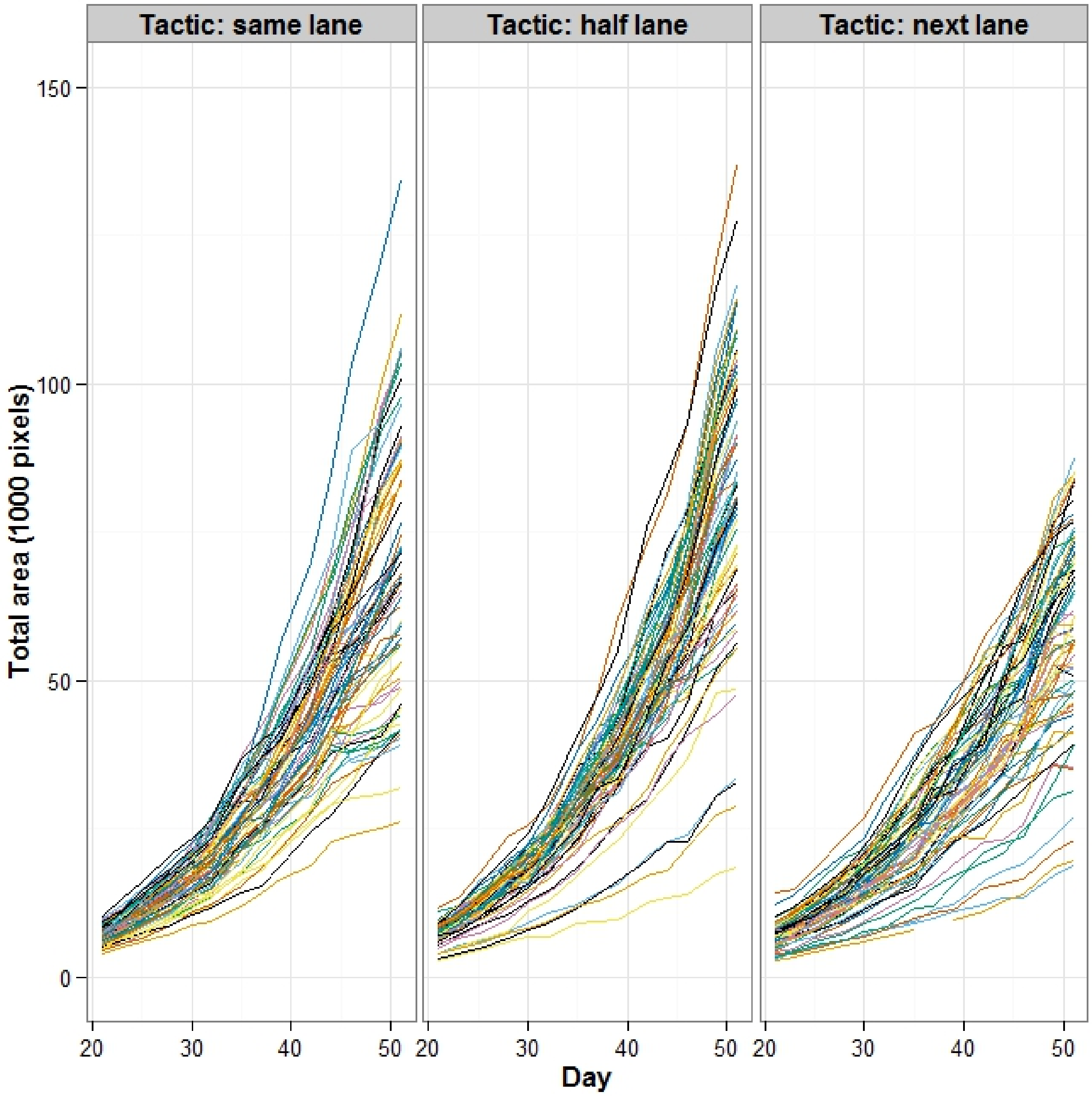

Figure 2 Trend in total area over time for individual plants for each tactic. The bench tactic is not included as it was only imaged in day 51. The trend over time shows a curved trend with variability between plants increasing over time. Next lane plants are on average smaller than those for the other tactics.
